# Potential of the Red Macroalga *Bonnemaisonia hamifera* in Reducing Methane Emissions from Ruminants

**DOI:** 10.3390/ani13182925

**Published:** 2023-09-15

**Authors:** Abdulai Guinguina, Maria Hayes, Fredrik Gröndahl, Sophie Julie Krizsan

**Affiliations:** 1 Department of Animal Nutrition and Management, Swedish University of Agricultural Sciences, 750 07 Uppsala, Sweden; sophie.krizsan@slu.se; 2 Animal Nutrition, Production Systems, Natural Resources Institute Finland (LUKE), 31600 Jokioinen, Finland; 3 Food BioSciences Department, Teagasc Food Research Centre, Ashtown, 15 Dublin, Ireland; maria.hayes@teagasc.ie; 4 Department of Sustainable Development, Environmental Science and Engineering, KTH Royal Institute of Technology, 100 44 Stockholm, Sweden; fredrik.grondahl@abe.kth.se

**Keywords:** dairy cow, greenhouse gas, macroalga, methane

## Abstract

**Simple Summary:**

Methane is a gas that ruminants naturally release during digestion, and it is a significant contributor to global warming. In efforts to reduce the environmental impact of livestock farming, we explored a red macroalga called *Bonnemaisonia hamifera*. This macroalga was collected from the shores of Sweden and used in an in vitro digestion experiment to evaluate its effects on ruminal fermentation and methane production from dairy cows. The study examined different inclusion levels of the macroalga in grass silage. We noticed an increase in the proportion of propionate in rumen fluid and a reduction in methane production with inclusion of the macroalga. This is important because reducing methane emissions from ruminants would be beneficial for the environment. *B. hamifera* exhibited antioxidant properties, which could be beneficial for the animals. In conclusion, this study shows that *B. hamifera* from Sweden has the potential to make livestock farming more eco-friendly by decreasing methane gas emissions.

**Abstract:**

Researchers have been exploring seaweeds to reduce methane (CH_4_) emissions from livestock. This study aimed to investigate the potential of a red macroalga, *B. hamifera*, as an alternative to mitigate CH_4_ emissions. *B. hamifera*, harvested from the west coast of Sweden, was used in an in vitro experiment using a fully automated gas production system. The experiment was a randomized complete block design consisting of a 48 h incubation that included a control (grass silage) and *B. hamifera* inclusions at 2.5%, 5.0%, and 7.5% of grass silage OM mixed with buffered rumen fluid. Predicted in vivo CH_4_ production and total gas production were estimated by applying a set of models to the gas production data and in vitro fermentation characteristics were evaluated. The results demonstrated that the inclusion of *B. hamifera* reduced (*p* = 0.01) predicted in vivo CH_4_ and total gas productions, and total gas production linearly decreased (*p* = 0.03) with inclusion of *B. hamifera*. The molar proportion of propionate increased (*p* = 0.03) while isovalerate decreased (*p* = 0.04) with inclusion of *B. hamifera*. Chemical analyses revealed that *B. hamifera* had moderate concentrations of polyphenols. The iodine content was low, and there was no detectable bromoform, suggesting quality advantages over *Asparagopsis taxiformis*. Additionally, *B. hamifera* exhibited antioxidant activity similar to Resveratrol. The findings of this study indicated that *B. hamifera* harvested from temperate waters of Sweden possesses capacity to mitigate CH_4_ in vitro.

## 1. Introduction

Over the past decade, the discussion on the negative impact of meat and dairy production on the environment has gained a considerable momentum due to methane (CH_4_) emissions and global warming. Globally, as much as 44% of the total CH_4_ emissions can be attributed to agriculture [1]. Approximately 40% of these emissions can be attributed to the fermentation of feed by cattle [2]. Research has demonstrated that the macroalga *A. taxiformis* is among the most effective feed additives for mitigating enteric CH_4_ emissions from ruminants [3,4]. The mechanism of reduction is largely attributed to halogenated secondary metabolites, particularly bromoform [3], which acts by directly inhibiting methanogenesis [5]. Researchers concluded that commercial production of *A*. *taxiformis* could create new economies due to the fact that small quantities of this seaweed in the diet of ruminant animals reduced CH_4_ emissions by up to 98% when included at 0.05% of organic matter (OM) intake [5]. However, bromoform is a known carcinogen, and there have been elevated concentrations of bromide and iodine in the milk of dairy cows fed with *A. taxiformis* [6,7]. Additionally, *A*. *taxiformis* is native to South Australia, and it is currently not cultivated in large quantities in the northern hemisphere. This has raised concerns about the feasibility of scaling up production and the potential for net CH_4_ reduction when supplementing ruminant diets with a cultivated tropical macroalga [8].

*B. hamifera* is also a type of red alga of the same order *Bonnemaisoniales* and family *Bonnemaisoniaceae* as *A. taxiformis*. In New Zealand *B. hamifera* was shown to have a strong CH_4_ inhibitory effect in vitro of 95.4%, and 98.8% relative to the basal feed substrate at inclusion levels of 6% and 10% on OM basis [9]. Furthermore, Mihaila et al. [8] showed that the primary bioactive compound bromoform in *A. taxiformis* was not detected in *B. hamifera*. We hypothesized that native, wild-harvested *B. hamifera* from the west coast of Sweden can display a CH_4_ inhibitory effect in vitro and be a temperate seaweed alternative for cultivation, and less susceptible to the loss of harmful volatile bioactives during processing and handling. The objective of this study was to measure the CH_4_ inhibitory effect in vitro of *B. hamifera* harvested in temperate waters of Sweden.

## 2. Materials and Methods

The macroalga *B. hamifera* was harvested from Kristineberg Center for Marine Research and Innovation in Fiskebäckskil (58°14′ N 11°27′ E) on the west coast of Sweden. The seaweed was harvested from the shore in accordance with the Nagoya protocol guidelines (https://www.cbd.int/abs/doc/protocol/nagoya-protocol-en.pdf, accessed on 7 August 2023), packed in cool boxes, and transported via overnight courier to Swedish University of Agricultural Sciences in Umeå on dry ice. Samples were washed to remove sand and epiphytes and stored at −18 °C. All samples were freeze-dried using a laboratory-scale Labconco FreeZone freeze dryer equipped with tray dryers (Labconco, Kansas city, MO, USA) operating at −84 °C.

The donor animals used for rumen inoculum, equipment used, and procedures of the in vitro experiment followed the recent work reported by Krizsan et al. [10]. In brief, rumen fluid was directly transported to the laboratory after collection and filtered through a cheesecloth into Thermos flasks. The samples were in total repeated across two water baths to get one bottle with blank (i.e., bottles with 60 mL of buffered rumen fluid with no sample or treatment within), duplicate bottles with control sample consisting of grass silage, and three replicates of treatment samples containing grass silage and *B. hamifera* in each bath. The *B. hamifera* was added at inclusion levels of 2.5%, 5%, and 7.5% on OM basis. All samples were randomly distributed among the in vitro bottles in each bath. Gas production was measured with a fully automated system (Gas Production Recorder, GPR-2, Version 1.0 2015, Wageningen UR, The Netherlands). Measurement of CH_4_ was performed by withdrawing gas samples (0.2 mL) at 2, 4, 8, 24, 32, and 48 h from all in vitro bottles. The concentration of CH_4_ was determined immediately after collection by injecting the gas sample in a Trace 1300 gas chromatograph (Thermo Fisher Scientific, Waltham, MA, USA) equipped with a split injector and a thermal conductivity detector (TCD). Separation was achieved using a 1.6 m packed column, using argon as the carrier gas with a flow rate of 32 mL/min and an isothermal oven temperature of 30 °C. A standard mixture of CO_2_ (900 mmol/mol) and CH_4_ (100 mmol/mol) was used as a calibration gas (AGA Gas AB, Sundbyberg, Sweden), and gas sample peaks were recognized by comparison with the standard gas. The CH_4_ concentration (mL/g sample) of all samples were used in model simulations to achieve in vivo predicted CH_4_ according to Ramin and Huhtanen [11].

For the alga, the N percentage in the sample was determined using the LECO FP628 (LECO Corp., St. Joseph, MI, USA) protein analyzer applying the Dumas AOAC method 992.15 (1990) [12] and protein content was obtained using a conversion factor of 5.0 [13]. The NDF concentration was determined free of residual ash following the protocol outlined by Van Soest et al. [14], using a 1020 hot and 1021 cold extractor (Tecator Fibertec System, FOSS Analytical AB, Höganäs, Sweden) with addition of heat-stable α-amylase and sodium sulphite. The percentage lipid in each sample was assessed using the Oracle NMR Smart Trac rapid Fat Analyzer (CEM Corporation, Matthews, NC, USA) using AOAC official methods 985.14. The ash and moisture contents were determined according to [12].

As detailed in Krizsan et al. [10], the total polyphenol concentration (TPC) of the macroalga was estimated using the Folin–Ciocalteau reagent (Sigma-Aldrich, St. Louis, MI, USA); the iodine content was determined using the Iodine Colorimetric Assay Kit (BioVision, Milpitas, CA, USA), and the antioxidant capacity was determined using the 2,2-diphenyl-1-picrylhydrazine (DPPH) Antioxidant Assay Kit (AbCam, Amsterdam, The Netherlands -ab289847, K2078). Resveratrol was used as a reference standard. Bromoform concentration in macroalga extract was carried out as described in Krizsan et al. [10].

Individual volatile fatty acid (VFA) concentrations in in vitro rumen fluid samples were determined using a Waters Alliance 2795 UPLC system (Waters Corporation, Milford, MA, USA) as described by Puhakka et al. [15]. In brief, rumen fluid was subjected to filtration using a 0.22 μm filter to remove any particulate matter. A 150 μL portion of the filtrate was diluted with an equal volume (150 μL) of 2-ethylbutyric acid (internal standard) in acetonitrile. In a sample vial, 40 μL of a 100 mM pentafluorobenzylhydroxylamine solution in water–acetonitrile (1:1) was added to 20 μL of the diluted sample. The contents in the vial were vigorously shaken for 5 s using a vortex shaker followed by an addition of 40 μL of a 250 mM activation reagent, specifically [1-ethyl-3-(3-dimethyl-aminopropyl)] carbodi-imide in ethanol containing 3% pyridine. The reaction vial was then heated for 60 min at 60 °C. Liquid chromatographic analysis was carried out with a detection wavelength set at 269 nm.

All statistical analyses were performed using SAS version 9.4 (SAS Institute Inc., Cary, NC, USA). Data were subjected to ANOVA using the MIXED procedure in SAS with treatment, water bath, and their interaction as fixed effects and bottle position in water bath as a random effect.

Treatments were compared using orthogonal contrasts; contrasts were constructed to evaluate the effects of inclusion of *B. hamifera*, and the linear and quadratic effects of inclusion levels.

## 3. Results

The macroalga had DM, OM, CP, and crude fat concentrations of 152 ± 1.3 g/kg of fresh weight, and 505 ± 6.7, 97 ± 3.1 and 4.3 ± 0.26 g/kg of DM.

**Figure 1:**
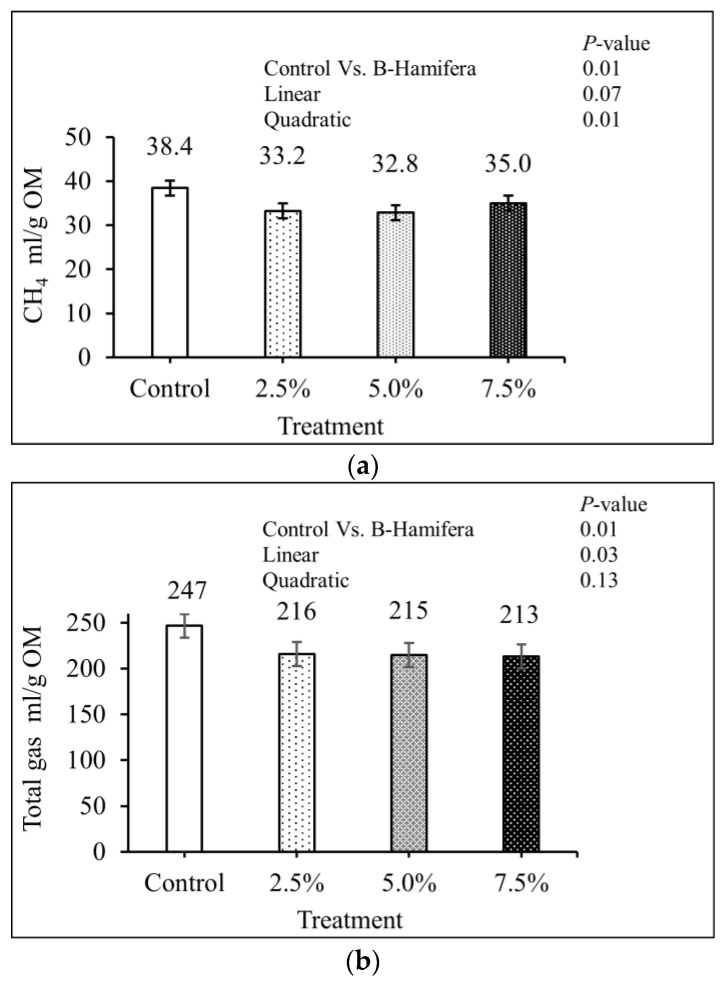
The effect of *B. hamifera* at different inclusion levels on predicted in vivo methane production (**a**) and total gas production (**b**) with SEM of 1.68 and 13.1 mL/g OM, respectively.

The inclusion of *B. hamifera* decreased (*p* = 0.01) predicted in vivo CH_4_ (Figure 1a) and total gas productions (Figure 1b). There was a quadratic effect (*p*  =  0.01) of increased levels of *B. hamifera* on predicted in vivo CH_4_ production. The predicted in vivo total gas production linearly decreased (*p*  =  0.03) due to higher total gas from control compared to the macroalga treatments.

Propionate was higher (*p* = 0.03) and isovalerate was lower (*p* = 0.04) with the inclusion of *B. hamifera* compared to the control (Table 1). A tendency of increased (0.06 ≤ *p* ≤ 0.10) total VFA production and proportions of butyrate, isobutyrate, and 2-methylbutyrate were observed with the inclusion of *B. hamifera* compared to the control. We also found a quadratic effect (*p*  ≤  0.05) on proportions of isobutyrate, 2-methylbutyrate, and isovalerate as well as a quadratic tendency (0.08 ≤ *p* ≤ 0.10) on total VFA production and the proportion of butyrate with an increase in *B. hamifera* inclusion levels. 

The respective average polyphenol and iodine contents of *B. hamifera* sample were 0.165 mg gallic acid equivalents and 71.1 µg/L iodine. The value obtained for total antioxidant activity of *B. hamifera* was 0.395 µM Trolox equivalents mg/mL sample. This is comparable to the reference standard Resveratrol, which had a DPPH value of 0.409 µM Trolox equivalents mg/mL (n = 3). There was no bromoform detected in the *B. hamifera* used in this study.

## 4. Discussion

The potential of feeding red algae to reduce CH_4_ emissions from ruminants is a promising solution for a more sustainable production of food from cattle. However, there needs to be a system for use, i.e., cultivating, distributing, and storing red algae on the farm without a change in the active substances occurring and assuring safety. The Primary goal is to guarantee an efficient CH_4_ mitigation, but it is equally important to minimize the harmful risk of substances like bromoform. Poor mixing and an accidently large dose of *A. taxiformis* could cause damage to the rumen wall of individual cows [16] and lead to reduced feed intake [6].

It is worth noting that a high concentration of bromoform in red algae have led to greater CH_4_ reduction [5]. In our study, the inclusion of *B. hamifera* resulted in a modest 12.3% reduction in predicted in vivo CH_4_ production compared to an earlier in vitro study conducted in New Zealand that reported CH_4_ reductions of at most 98.8% at an inclusion level of 10% on an OM basis [9]. The inhibitory effect seemed to be mediated by longer-chained halogenated hydrocarbons, likely by the same inhibitory mechanism as *A. taxiformis* [9]. Enge et al. [17] found that *B. hamifera* produced 1,1,3,3-tetrabromo-2-heptanone (a halogenated secondary metabolites) as a chemical defense and as the main feeding deterrent compound. This compound could be a prospective candidate for exhibiting anti-methanogenic effect in the rumen.

In terms of ruminal fermentation patterns, most in vivo experiments with red algae have demonstrated a shift towards increased propionate production, confirming its role in CH_4_ inhibition [5,6,7,18]. However, the increase in molar proportion of propionate with *B. hamifera* inclusion was generally small in this study and likely of minor biological relevance. Several CH_4_ inhibitory mechanisms could have been the reason for the effect observed in the present study, but most likely the bioactive substances in Swedish *B. hamifera* affected a broader spectrum of the microbiome since total gas was decreased in supplemented treatments in vitro. Depending on where they grow and when they are harvested, algae will contain different levels of bioactive substances [13], which likely can explain the observed differences between *B. hamifera* harvested in Sweden and New Zealand. Ruminal branched-chain VFA (BCVFA; isobutyrate, isovalerate, and 2-methylbutyrate) are derived mainly from the deamination of branched-chain amino acids in the diet. Branched-chain VFA supplementation has been shown to improve digestibility and production in ruminants by providing an additional energy source and promoting the proliferation of cellulolytic bacteria [19]. In our study, the reduction in BCVFA proportions may indicate less microbial activity, contributing to the overall reduction in CH_4_ emissions.

In many ways, red algae open up the possibility of producing organic food from dairy cows with reduced CH_4_ emissions. However, *B. hamifera* harvested on the west coast of Sweden does not provide a satisfactory reduction of CH_4_ compared to other more readily available dietary mitigation strategies that could be suitable also in organic cattle production. On the other hand, the low iodine content and absence of bromoform in *B. hamifera* make it a potentially safer and more environmentally friendly option compared to *A. taxiformis* for CH_4_ mitigation in ruminants. These characteristics reduce the risk of negative health effects on animals and minimize potential ecological concerns. However, further research is necessary to fully understand the specific bioactive substances present in *B. hamifera* and their effects on CH_4_ production to optimize its utilization as a sustainable solution for reducing greenhouse gas emissions in livestock production. To further understand the differences observed in CH_4_ inhibition, it is important to investigate the conditions specific to New Zealand, where more significant reductions in CH_4_ emissions were reported in previous studies.

## 5. Conclusions

Results from the current study showed that *B. hamifera* supplementation led to a modest reduction (12.3%) in predicted in vivo methane production, suggesting its potential as a sustainable strategy for reducing greenhouse gas emissions in cattle production. However, the observed effect on ruminal fermentation patterns was relatively small and may have minimal biological importance. Additionally, the absence of bromoform and the low iodine content in *B. hamifera* make it a safer and more environmentally friendly option compared to some other red algae species.

## Figures and Tables

**Table 1:** Effects of *B. hamifera* at different inclusion levels on total volatile fatty acid (VFA) and molar proportions of VFA production at 48 h of incubation in vitro.

Item	Treatments				SEM	*p*-Value		
	Control	*B. hamifera* Inclusion Level (% OM)						
		2.5%	5.0%	7.5%		Control vs. *B. hamifera*	Linear	Quadratic
Total VFA, mM	148	162	159	155	6.6	0.10	0.44	0.08
VFA molar proportions, mmol/mol								
Acetate	575	574	575	577	2.4	0.94	0.39	0.42
Propionate	241	246	244	245	2.2	0.03	0.11	0.13
Butyrate	98.4	96.7	96.9	97.5	0.90	0.10	0.39	0.09
Isobutyrate	14.9	14.2	14.2	14.6	0.34	0.06	0.38	0.04
2-Methylbutyrate	11.6	10.9	11.0	11.2	0.31	0.06	0.36	0.05
Isovalerate	13.8	12.8	13.0	13.3	0.39	0.04	0.33	0.03
Valerate	24.5	23.8	23.8	19.9	3.49	0.52	0.24	0.53
Caproate	21.2	21.9	21.3	21.6	0.40	0.29	0.62	0.54

## Data Availability

The data presented in this study are available on request from the corresponding author.
